# Probing E/Z Isomerism Using Pillar[4]pyridinium/Gold Nanoparticle Ensembles and Their Photoresponsive Behavior

**DOI:** 10.1021/acs.langmuir.2c00342

**Published:** 2022-04-15

**Authors:** Mykola Kravets, Iwona Misztalewska-Turkowicz, Volodymyr Sashuk

**Affiliations:** † Institute of Physical Chemistry, Polish Academy of Sciences, Kasprzaka 44/52, 01-224 Warsaw, Poland; ‡ Faculty of Chemistry, University of Bialystok, Ciołkowskiego 1K, 15-245 Bialystok, Poland

## Abstract

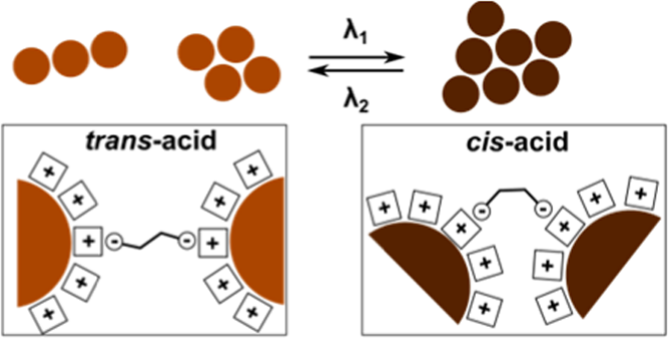

Despite the fundamental importance and broad applicability of E/Z dicarboxylic acids, their discrimination remains challenging and greatly unexplored. Herein, we present a general approach for the recognition of E/Z diacids using supramolecular interactions coupled with plasmonic response. The method allows detecting both single isomers and their light-induced interconversion, which ultimately entails multiple reversible nanoparticle aggregations. Such a molecular recognition-coupled responsive nanoscale self-assembly resembles natural mechanisms and can be a versatile means of building artificial complexity.

## Introduction

**Figure 1:**
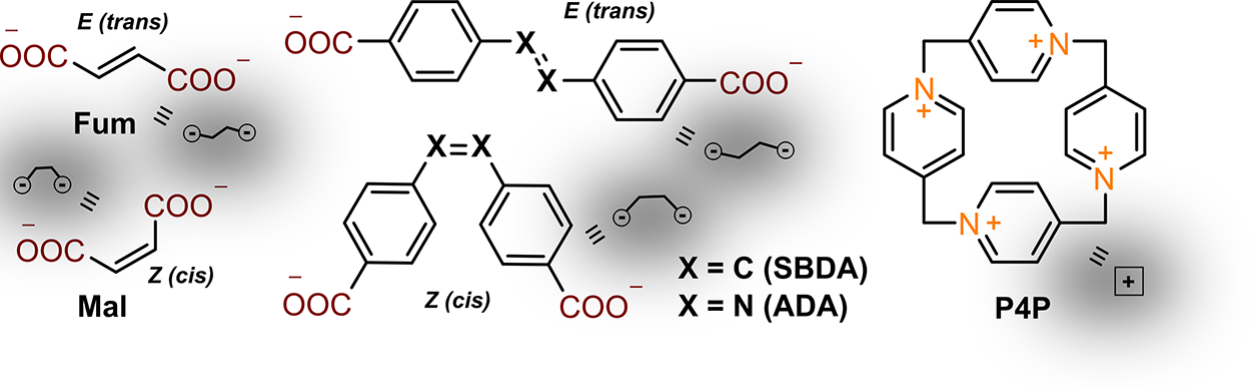
Dicarboxylates and pillar[4]pyridinium used in the present study.

Geometric isomers, emerging from the restricted rotation around a double X=X bond (where X is either C or N), are important compounds, relevant to many fields. Butenedioic acid is arguably the most recognizable compound among them. Its *trans* (E) form (fumaric acid, Fum) is ubiquitous in nature, e.g., as the intermediate in the Krebs cycle, whereas the *cis* (Z) isomer (maleic acid, Mal) is abiotic; both are indispensable for the food industry, pharmaceutics, and so on (Figure 1). Other prominent examples are azobenzenes and stilbenes, whose photoswitchable behavior is widely utilized in responsive systems.^1^ In particular, their dicarboxylic derivatives (azobenzene-4,4′-dicarboxylic acid (ADA) and stilbene-4,4′-dicarboxylic acid (SBDA), Figure 1) can reversibly regulate mechanical properties of thin films,^2^ macroscale motion of MOFs,^3^ hydrogel self-healing,^4,5^ molecular self-assembly,^6,7^ selective gas uptake^8^ and sieving,^9^ crystal growth,^10^ enzyme-like catalysis,^11^ and drug delivery and release.^12^

Given the similar appearance but distinct functions of these acids, the spatiotemporal differentiation of each is of vast importance for their proper use and effectiveness. Despite several reports on detecting butenedioic acid,^13−21^ a general strategy for the recognition of E/Z diacids has not yet been developed.

Recently, we presented a nanoplasmonic platform^22,23^ for probing various types of chemical similarity. We demonstrated that small differences in the structure can be revealed using gold nanoparticles covered with cationic pillar[*n*]pyridinium macrocycles.^24,25^ In the presence of carboxylic diacids, the nanoparticles approach one another by supramolecular interactions, resulting in plasmonic coupling, whose strength is proportional to the relative distance between carboxylic groups. In this manner, we were able to discriminate positional isomers^22^ and homologous carboxylic diacids.^23^ Herein, we report the differentiation of geometric isomers. The method allows for the recognition of both single isomers and their multiple light-induced interconversion, which, apart from the optical response, leads to repetitive nanoparticle assembly and disassembly, representing a new promising direction for the utilization of geometric isomers.

## Experimental Section

### General Information

Mal, Fum, and *tran*s-SBDA are used as received from commercial suppliers. *Tran*s-ADA was synthesized according to the literature protocol.^26^*Cis*-ADA and *cis*-SBDA were generated from *trans* forms of these acids by irradiation. All experiments were performed at room temperature in the dark. Solvents were of analytical grade quality. Deionized water (18.3 MΩ·cm) was obtained from a Milli-Q station. UV–Vis spectra were recorded using an Evolution220 spectrophotometer from Thermo Scientific. DLS and ζ-potential were measured on a Malvern Zetasizer. NMR spectra were recorded on a 400 MHz Varian instrument. TEM images were taken with an FEI TECNAI and analyzed using ImageJ. pH was measured using a HI 3220 pH meter equipped with an InLab Micro glass electrode (Mettler Toledo).

### Synthesis of “Naked” Gold Nanoparticles (AuNPs)

To 10 mL of water was added 495 μL of 24.3 mM aqueous HAuCl_4_ solution. After 4 min of stirring, to this mixture was added 600 μL of 100 mM freshly prepared aqueous NaBH_4_ solution by a single injection. The final solution was stirred for 2 min and left for 2 days to allow unreacted BH_4_^–^ ions to decompose.

### Synthesis of P4P-Coated Gold NPs

To 100 μL of 58.82 mM aqueous pillar[4]pyridinium (P4P) solution, 1200 μL of 1.08 mM AuNPs (in terms of gold atoms) was added portionwise at 200 μL (each portion was added about every 10 s) with constant stirring for 2 min. The obtained solution was immediately used further.

### Adjustment of pH

The pH of the P4P-coated gold NP solution was measured as 3.2. To increase pH, small portions of aq. NaOH (0.1 M) were added until the desired acidity was achieved.

### Acid Sensing

The P4P-coated gold NP solution was divided into 300 μL portions. To each was added a single acid (200 μL), in the form of sodium salt, 11 mM in the case of fumaric and maleic acids, 0.433 mM in the case of ADA and SBDA, and 0.011 M in the case of acetic acid. Accordingly, blank solutions were diluted with the same volume of pure water. The resultant solutions were then transferred—in whole or in part (depending on the required volume)—either into a quartz cuvette (UV–Vis measurements) or in a polystyrene cuvette (DLS measurements) or in a folded capillary cell (ζ-potential measurements) or were deposited and dried on TEM grids for TEM analysis.

### UV–Vis Measurements

The P4P-coated gold NP solutions mixed with diacids were transferred in 300 μL quartz cuvettes, and the spectra were recorded.

### DLS Measurements

A total of 500 μL of the resultant solutions was transferred to 3 mL polystyrene cuvettes and diluted threefold. The average size of the particles (the volume-weighted average value) was recorded for gold nanoparticles covered with P4P before and after the addition of the dicarboxylic acids.

### ζ-Potential Measurements

A total of 500 μL of the resultant solutions was diluted twice and transferred to 1 mL folded capillary cells. The charge of the particles was recorded for gold nanoparticles covered with P4P before and after the addition of the dicarboxylic acids.

### TEM Analysis

A total of 30 μL of the resultant solutions was diluted 25 times, and 1 μL of the final solutions was deposited and dried on TEM grids for further analysis.

### Photoisomerization Experiments

The samples were irradiated with 254, 350, and 430 nm monochromatic light using a xenon arc lamp with a bandwidth of 20 nm installed in an RF 6000 fluorometer at a distance of 5 cm for 10 min.

## Results and Discussion

As in our previous work,^23^ we employed ∼4.5 nm gold nanoparticles prepared by the reduction of chloroauric acid with sodium borohydride. Among available pillar[*n*]pyridiniums, we chose the smallest one (P4P, Figure 1) due to its larger sensitiveness toward diacids.^23^ To compile the sensor, both components were brought together to yield a colloid with C(P4P) = 4.5 mM and C(AuNPs) = 1 mM (in terms of gold). The excess of the macrocycle was necessary for the stabilization of the resultant colloid. The colloid was not buffered due to the aggregation of the NPs. The NP concentration was taken to be detectable by UV–Vis spectroscopy. The acids were used in the form of sodium salts.

**Figure 2:**
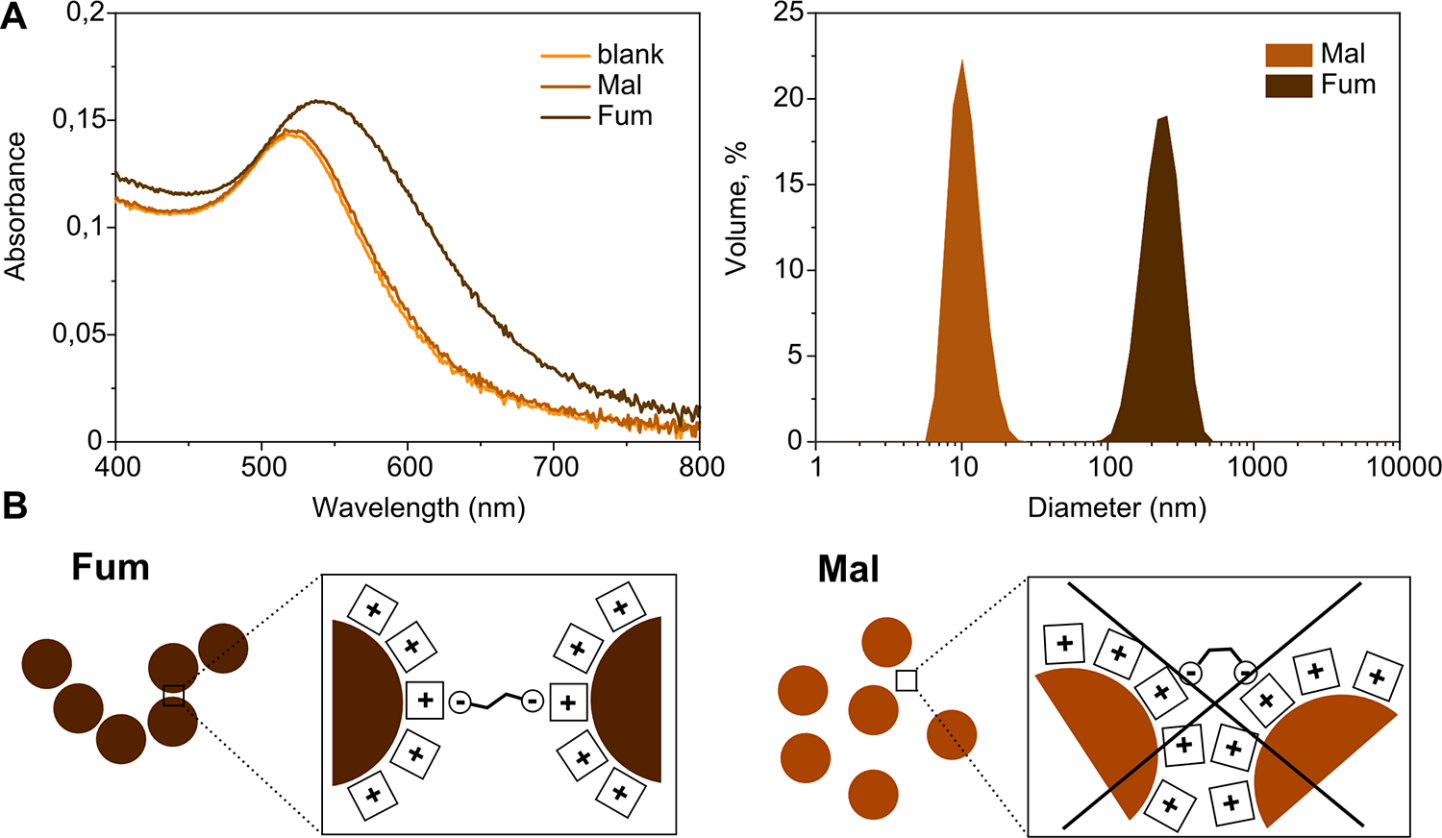
(A) Absorption spectra and average particle size of gold nanoparticles 10 min after the addition of maleic and fumaric acids. (B) Plausible interaction patterns (or lack thereof) in the presence of the abovementioned acids.

The study was begun by testing the isomers of butendioic acid. To receive a perceivable colorimetric response, the acids were employed in a sevenfold excess (C(P4P) = 2.7 mM, C(AuNPs) = 0.6 mM, C(acid) = 4.5 mM). Only the *trans* (fumaric, Fum) form gave a strong signal (the sample turned violet, Figure S1), shifting the plasmon band to the red region (+25 nm) and increasing its intensity (+10%), indicating NPs approaching and plasmon coupling (Figure 2A). Evidently, this was made possible due to the opposite directionality of carboxylic groups that could interact with positively charged P4P on the nanoparticle surface (Figure 2B). These interactions induced multiple NP interconnections and, in a short time (10 min), the formation of sizable 220 nm aggregates, as revealed by DLS. On the contrary, the *cis* (maleic, Mal) isomer, whose carboxylic groups lie on the same side of the C=C bond, is not able to establish an effective connection between the NPs, as this would require an approach at a distance less than the diameter of a single particle. Indeed, the plasmon band of the sample changed only a little (+8 nm, the color remained red, as seen by the naked eye), and DLS showed almost no increase in the NP size (10 nm). Markedly, little change in plasmon response was also observed in the presence of monocarboxylic (acetic) acid (C = 4.5 mM, Figure S2), indicating the necessity of two opposite carboxylic groups for efficient NP cross-linking.

**Figure 3:**
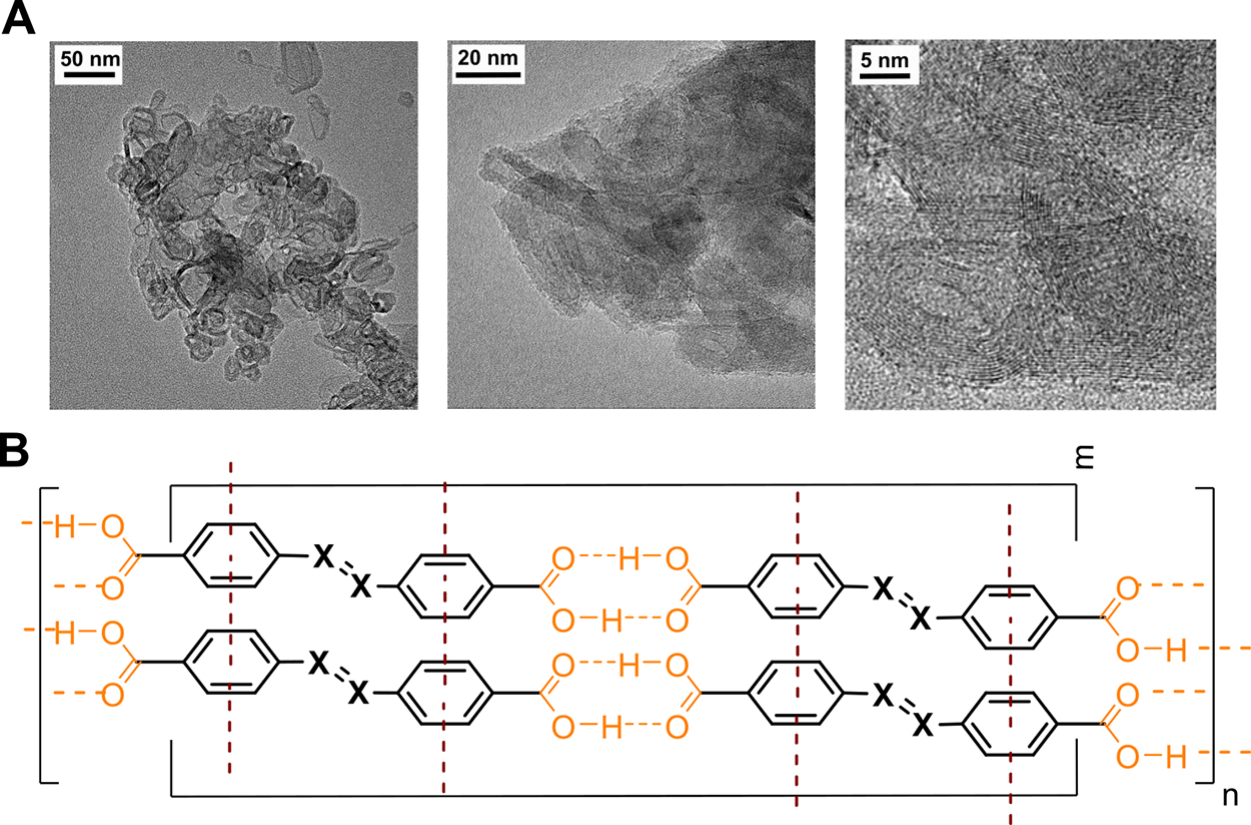
TEM images of a wool-like material precipitated from aromatic acid samples (herein *trans*-ADA) at low pHs (A) and its plausible chemical structure (B).

Surprisingly, a completely different response, which required as little as 0.9 equiv of acid, was brought about by aromatic acids (ADA and SBDA). The sedimentation of the cotton-like red precipitate (plasmon band centered at ∼525 nm) was observed instead regardless of whether the acids were in a *trans* or *cis* configuration. A TEM examination of the precipitate revealed microns long folded ribbons, each consisting of over a dozen filaments with an average thickness of 1.7 Å (Figures 3A and S3), and very rare nanoparticles around (not shown). This microscopy picture explained the bare color change (no plasmon coupling between the NPs) and indicated the presence of an organic material. The formation of the latter, considering the thickness of the single filaments, seems to be due to the hydrogen bond-mediated self-assembly of protonated ADA and SBDA acids and π–π stacking of the resultant polymeric chains (Figure 3B).^27^

**Figure 4:**
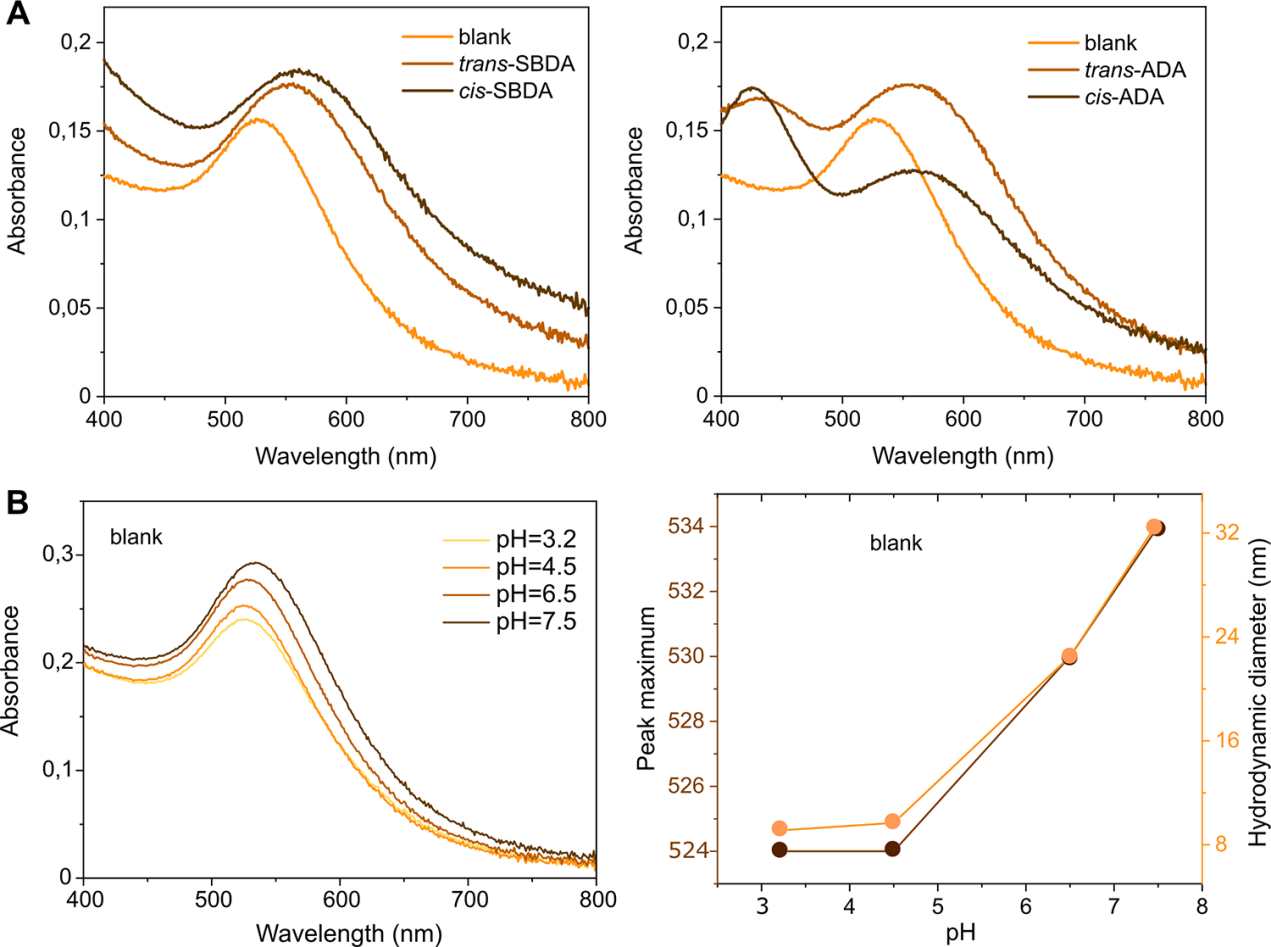
(A) Absorption spectra of gold nanoparticles 10 min after the addition of isomeric SBDA and ADA. For the spectra at shorter times, see Figure S4. (B) Absorption spectra of the P4P-containing gold colloid at different pH values and plotted against their λ_max_ and hydrodynamic size.

To avoid acid protonation and precipitation, we increased the pH of the AuNP feed solution from 3.2 to 4.25. This ultimately enabled the discrimination of ADA and SBDA acids (Figure 4A). Expectedly, *cis* isomers, due to the shorter distance between the carboxylic groups, induced larger plasmon band shifts than *trans* ones (*cis* 558 nm vs *trans* 554 nm for ADA, *cis* 558 vs *trans* 551 nm for SBDA). Moreover, much larger shifts than previously observed were noticed for Fum (+50 nm) and Mal (+20 nm) samples (Figure S5). This indicated that in addition to the “specific” aggregation, i.e., evoked by NP cross-linking, there is a “nonspecific” one resulting from the positive surface charge neutralization and NP attraction as pH increases. This type of aggregation can be especially clearly seen in the neat colloid containing no E/Z acids (Figure 4B); however, its contribution to the overall aggregation is not large. The crucial role in NP aggregation is played by the dicarboxylic acids. This is nicely exemplified by the plasmon shifts of butendioic acid samples described above. The Mal sample, despite the higher final pH (6.59), aggregates to a much lower extent than the Fum sample (pH = 5.35).

The aggregation of SBDA and ADA samples was further supported by DLS. The average size of “Z-aggregates” was found to be larger than the size of “E-aggregates”, 340 and 150 nm for ADA and 450 and 270 nm for SBDA. The differences in sizes come likely from the better surface charge neutralization by *cis* isomers than *trans* ones. This was corroborated by ζ-potential measurements, giving 15.7 mV for the sample with *trans*-ADA and 14.4 mV for the NPs containing the *cis* form. Considering the facile isomer interconversion, we thought to apply these changes in reversible NP self-assembly. Previously, the latter was usually done by covalent attachment of molecular photoswitches to the NP surface.^28^ P4P-AuNP ensembles open up the opportunity to perform the process noncovalently, and therefore much easier, using a photoresponsive medium.^29−32^

**Figure 5:**
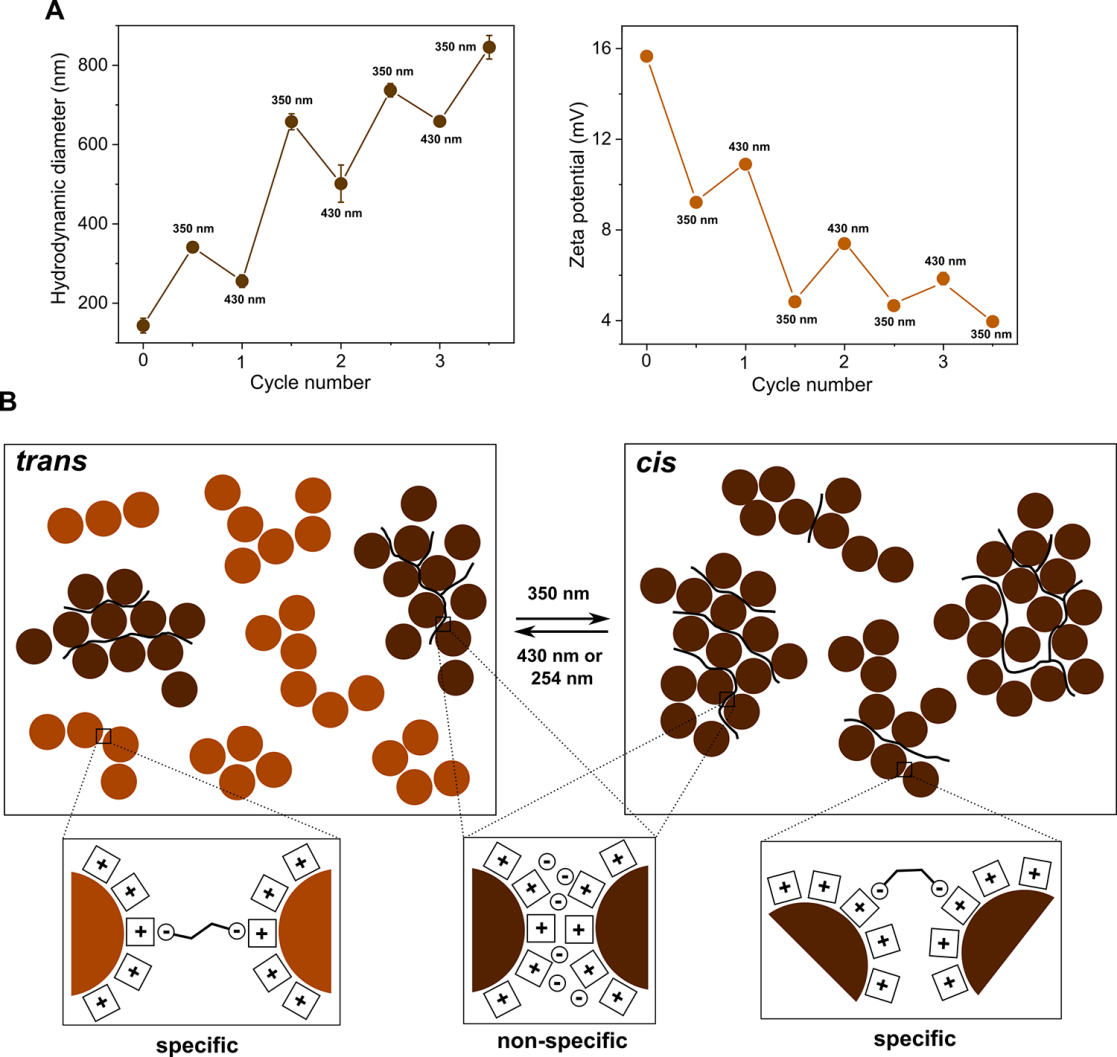
(A) Changes in average particle size and ζ-potential of *trans*-ADA samples under alternating ultraviolet and blue light irradiation and (B) putative mechanism of NP reversible aggregation for ADA and SBDA samples (black lines within the aggregates mark regions of nonspecific interactions).

To implement this idea, we had to stabilize the NPs. To this end, we diluted the colloidal solutions threefold (C(AuNPs) = 0.2 mM, C(acid) = 0.18 mM). This slowed down the aggregation rate appreciably. By exposing the *trans* samples to alternating irradiation, first UV (350 nm) and then visible light (430 nm for ADA) or UV (254 nm for SBDA), we could assemble and disassemble the NPs into larger and smaller aggregates at least three times in a row (Figures 5A and S6—DLS; Figures S7 and S8—TEM). UV–Vis and NMR spectra of ADA and SBDA before and after irradiation are shown in the Supporting Information (Figures S9 and S10). Interestingly, after the first cycle, the average aggregate size increased; then, depending on the diacid used, it remained steady (SBDA) or increased further (ADA), which indicated unequal contributions of “nonspecific” aggregation. This was supported by pH measurements of the final solutions, showing the less-acidic character of the ADA sample (6.88) compared to the SBDA one (6.69). The destabilization and partial temporal restoration of colloidal stability of the ADA sample upon light switching are also nicely seen from ζ-potential experiments (Figure 5A). Importantly, the NPs could be similarly toggled back and forth, starting not only from *trans* but also the *cis* configuration (Figures S11 and S12). This demonstrates the high versatility of the system.

## Conclusions

In summary, we have demonstrated that simple pillar[4]pyridinium/gold nanoparticle ensembles can be utilized for the recognition of E/Z dicarboxylic acids. The process can be followed by the changes in the optical density, charge, and dispersion state of the NPs. The occurrence and magnitude of each kind of response depend primarily on the spatial position and relative distance between the carboxylic groups. For short diacids, the more pronounced response is obtained for *trans* isomers, whereas, for long acids, *cis* forms come to the fore. Remarkably, when the acids are prone to facile isomer interconversion, the changes in the charge and dispersion state of the NPs become partially reversible. This allows for the repeatable recognition of each isomer and, in parallel, for the creation of the responsive colloidal system. Upon alternating wavelengths of light, the NPs that constitute this system undergo multifold assembly and disassembly. Such a combination of molecular recognition and responsive self-assembly resembles natural processes and offers a straightforward route for achieving dynamic chemical complexity in artificial systems.

## Supporting Information Available

Photographs of the macroappearance of the samples, UV–Vis and NMR spectra, DLS and ζ-potential data, and TEM micrographs (PDF)
