# Supplementary material for: Probing E/Z Isomerism Using Pillar[4]pyridinium/Gold Nanoparticle Ensembles and Their Photoresponsive Behavior
Source: Langmuir. 2022 Apr 15;38(16):4942–7. doi: 10.1021/acs.langmuir.2c00342 (PMC9047399; doi:10.1021/acs.langmuir.2c00342)
Supplement: Supplementary file 1 — la2c00342_si_001.pdf [file la2c00342_si_001.pdf]

Supporting information

Probing E/Z Isomerism Using Pillar[4]pyridinium/Gold Nanoparticle Ensembles and Their Photoresponsive Behavior

Mykola Kravets,[†] Iwona Misztalewska-Turkowicz,[‡] and Volodymyr Sashuk[†]*

[†]*Institute of Physical Chemistry, Polish Academy of Sciences, Kasprzaka 44/52, 01-224 Warsaw, Poland*

[‡]*University of Bialystok, Faculty of Chemistry, Ciołkowskiego 1K, 15-245 Bialystok, Poland*

*E-mail: vsashuk@ichf.edu.pl

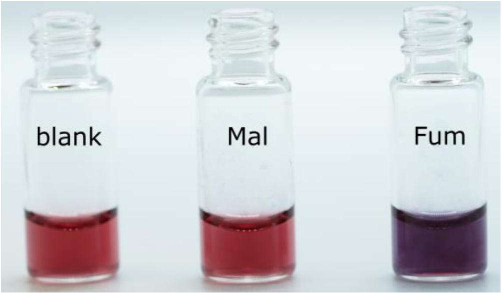

Figure S1. Photographs of gold NP solutions before and after addition of Mal and Fum acids.

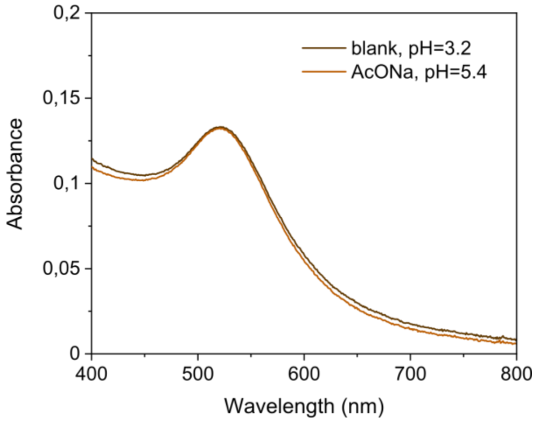

Figure S2. Absorbance spectra of P4P-containing AuNPs before and after the addition of AcONa.

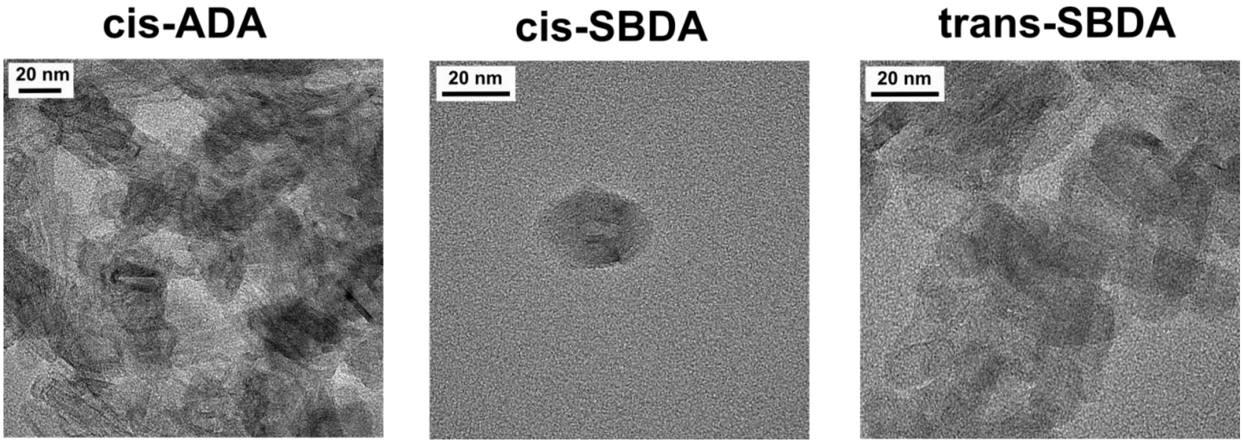

Figure S3. TEM micrograms of an organic material formed in aromatic acid samples.

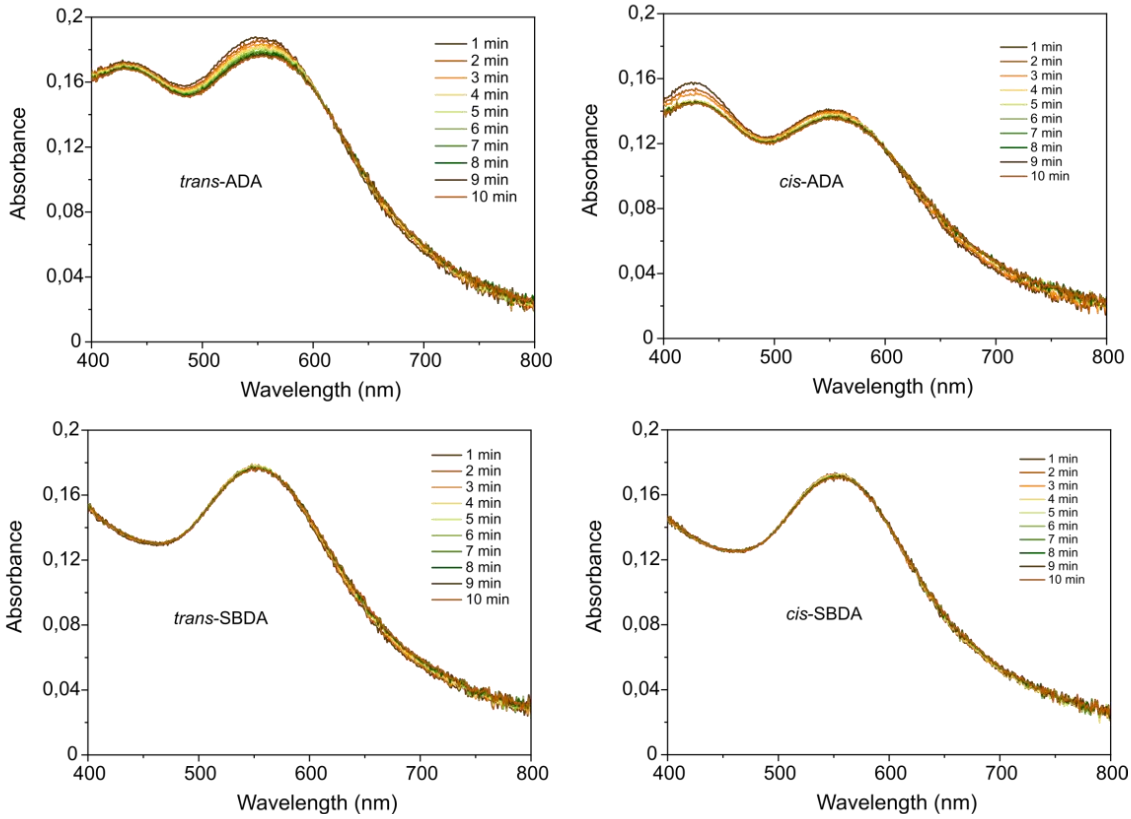

Figure S4. Absorbance spectra showing the stability of ADA and SBDA samples over time.

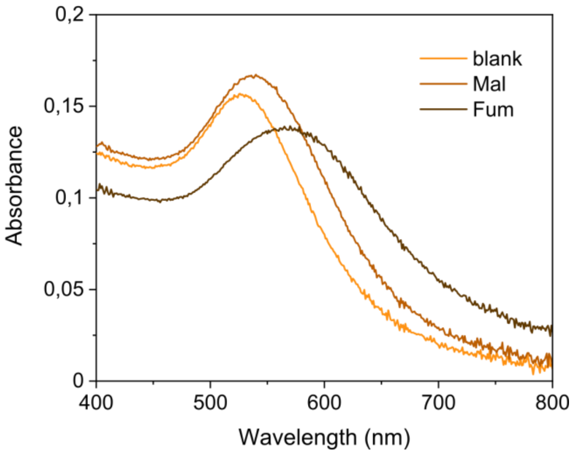

Figure S5. Absorbance spectra of Fum and Mal samples at higher pHs (5.35 for Fum and 6.59 for Mal).

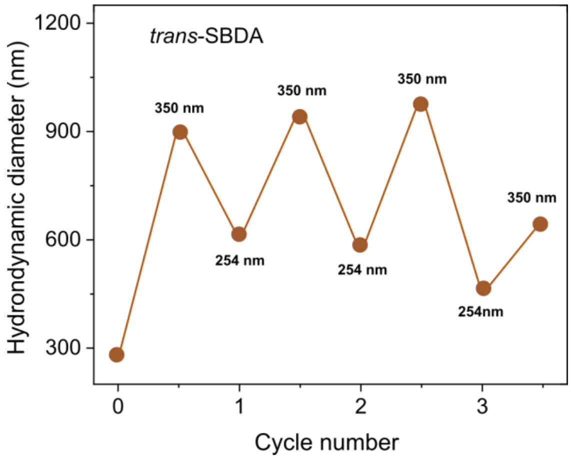

Figure S6. Photoswitching of average particle size in *trans*-SDBA sample.

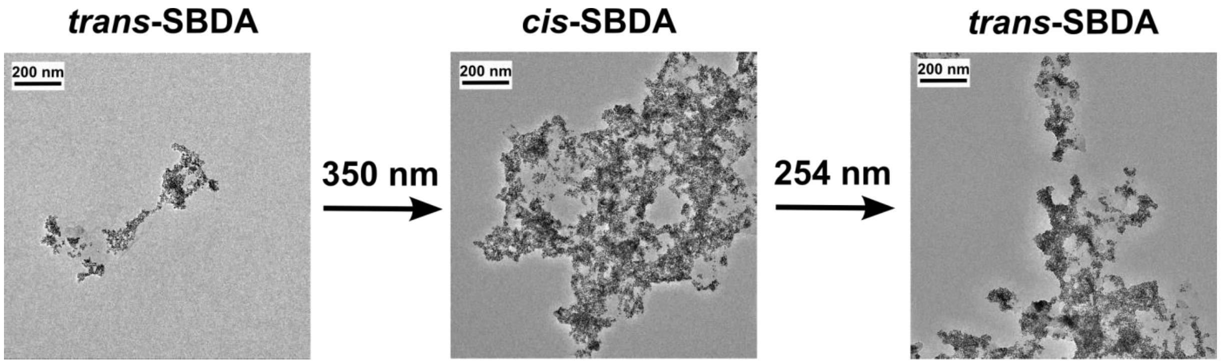

Figure S7. TEM micrographs of *trans*-SBDA sample after consecutive middle and near ultraviolet irradiation.

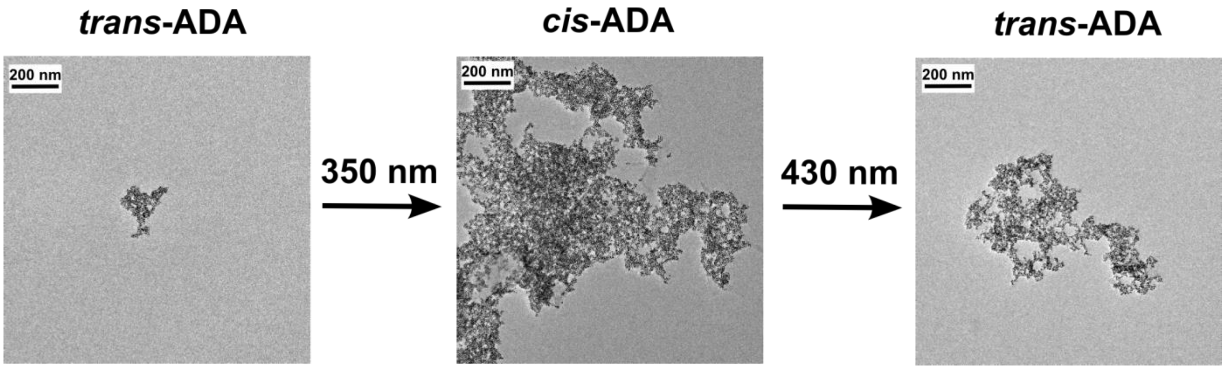

Figure S8. TEM micrographs of *trans*-ADA sample after consecutive ultraviolet and blue light irradiation.

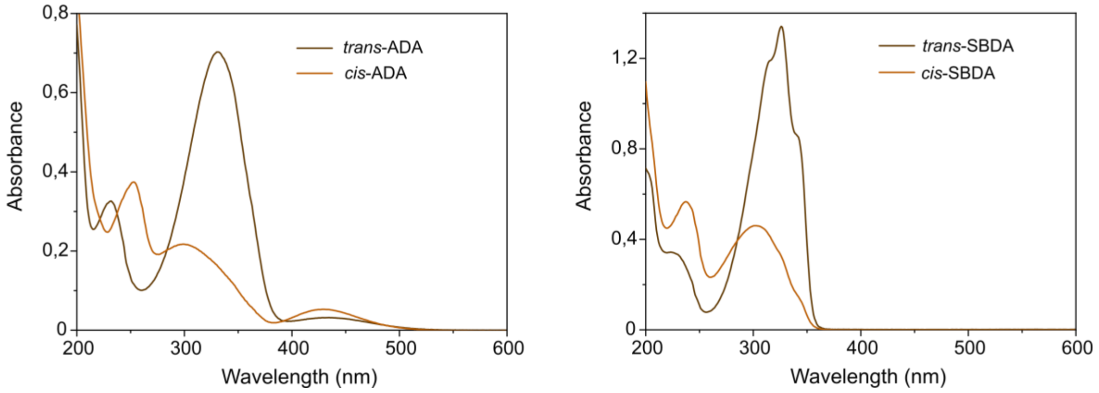

Figure S9. UV-Vis spectra of ADA and SBDA before and after irradiation. Note that the samples were irradiated until the spectra did not change.

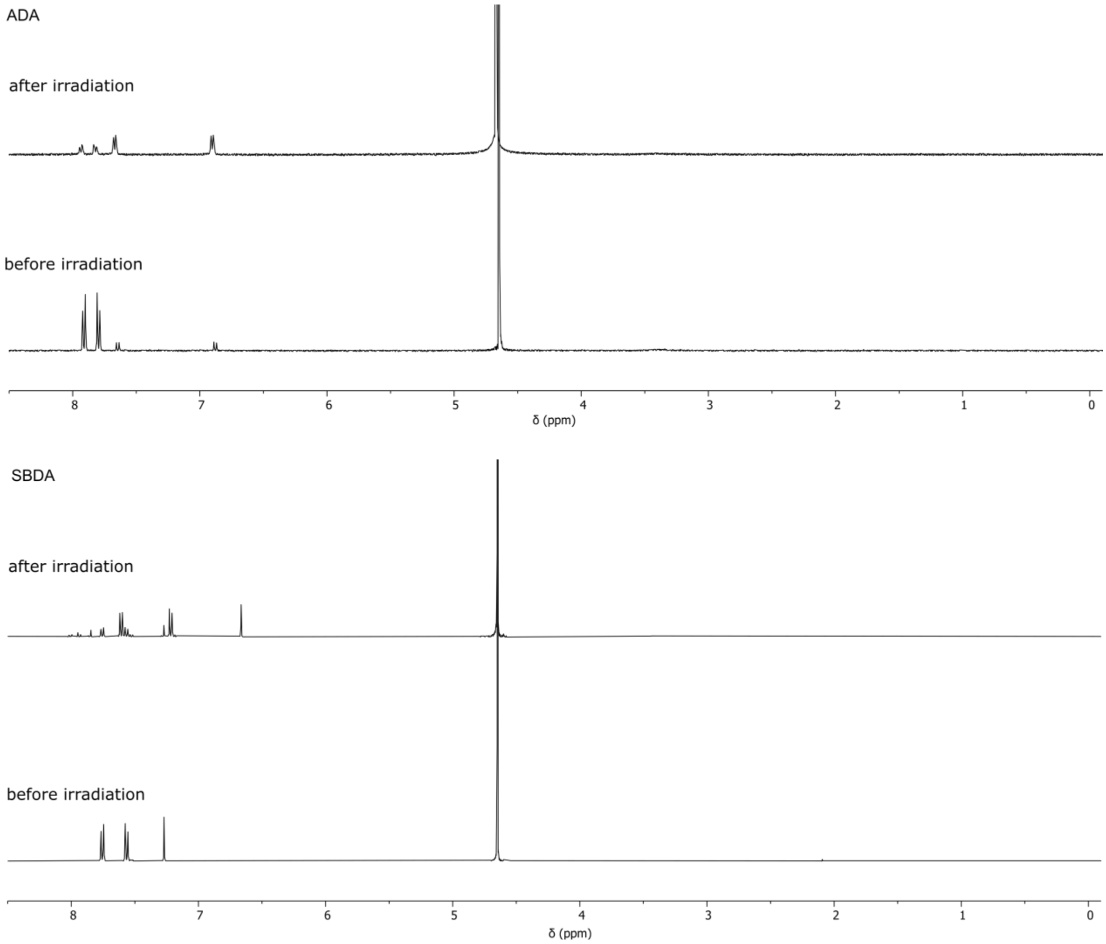

Figure S10. ^{1}H NMR spectra of ADA and SBDA before and after irradiation. The *trans*/*cis* isomer ratio was calculated as 88:12 for ADA and 100:0 for SBDA at the beginning of the experiment and 32:68 for ADA and 29:71 for SBDA at the end. Note that the actual content of *cis* isomers immediately after irradiation was probably higher as the NMR spectra were recorded ca. 10 min after completion of irradiation.

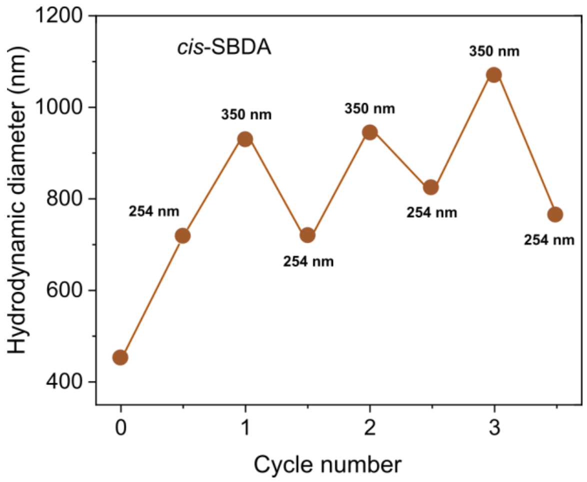

Figure S11. Photoswitching of average particle size in *cis*-SBDA sample.

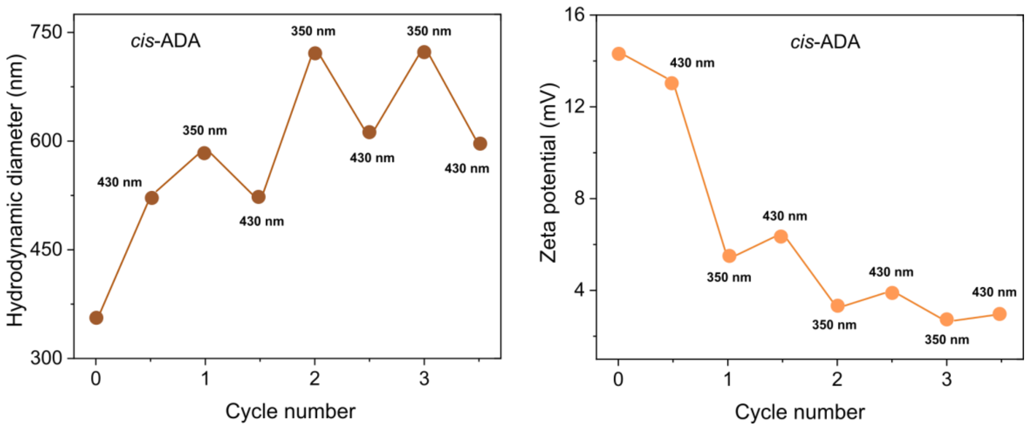

Figure S12. Photoswitching of average particle size and ξ-potential in *cis*-ADA sample.
